# Donor-derived infections in heart and lung transplant recipients

**DOI:** 10.1016/j.jhlto.2025.100376

**Published:** 2025-08-20

**Authors:** Bradley J. Gardiner, Michael G. Ison

**Affiliations:** a Department of Infectious Disease, Alfred Health, Melbourne, Victoria, Australia; b School of Translational Medicine, Monash University, Melbourne, Victoria, Australia; c Bethesda, MD

**Keywords:** lung transplant, heart transplant, donor-derived infection, emerging pathogens, transplant infectious disease

## Abstract

Heart and lung transplantation are life-saving treatments for patients with end-stage organ disease. Donor-derived infections are common and can be expected or unexpected. The lung is exposed to the external environment and transplanted with an intact microbiome, which can include community and nosocomial bacterial, viral, and fungal pathogens. This includes not only well-recognized scenarios, such as bacteria with or without multidrug resistance, respiratory viruses including SARS-CoV-2, molds, and tuberculous/nontuberculous mycobacteria, but also emerging pathogens, such as the mollicutes. The heart is the only transplanted organ that is a muscle and in direct contact with the bloodstream. These factors make donor-derived endocarditis, toxoplasmosis, and Chagas disease particularly relevant. This article aims to review some key established and emerging donor-derived infections that are of particular significance to heart and lung transplant recipients.

## Background

Solid organ transplantation offers a new lease on life for patients with end-stage organ disease but carries an inherent risk of donor-derived infection (DDI). Some of these are expected and have robust testing and prophylaxis strategies in place to reduce impact. Others are rare and unexpected and often are associated with serious consequences. In the modern era of increased global migration and travel, as well as an increasing number of transplants being performed globally, geographically restricted infections are becoming increasingly relevant for all recipients worldwide. Risk stratification of donors can be difficult due to the nature of the donor interview, with often incomplete information obtained in a time-sensitive fashion indirectly from donor relatives, who may not be familiar with all relevant history.

Recognizing DDI is crucially important as one donor can provide organs to up to 8 recipients, who may be geographically separated, so prompt identification and communication are crucial in mitigating impact.^1^ Ultimately, risk of DDI needs to be balanced against the risk of waitlist mortality, and an increased understanding of the potential pathogens and their consequences informs mitigation strategies and enables maximal utilization of the donor pool.^2^ The aim of this review is to outline some of the unexpected, rarer DDI that have a unique significance in heart and lung transplant recipients (Table 1).

**Table 1:** Summary of Several Key Donor-Derived Infections Relevant to Heart and Lung Transplant Recipients

Category	Examples	Key points
**Lung transplant recipients**		
Bacterial pathogens, including multidrug-resistant organisms	Methicillin-resistant *Staphylococcus aureus* Carbapenem-resistant *Enterobacteriaceae, Acinetobacter baumannii* Multidrug resistant *Pseudomonas aeruginosa*	• Respiratory cultures are commonly positive • Antimicrobial prophylaxis targeted to identified organisms can prevent early bacterial pneumonia • Donor–derived multidrug-resistant organisms more likely to fail conventional prophylaxis • With effective communication and targeted prophylaxis or pre-emptive therapy, transplantation can proceed safely with good outcomes • Balancing risks and benefits of antibiotics is crucial
Respiratory viral infections	SARS-CoV-2	• Donor screening recommended, with careful consideration to proceeding with lung transplantation based on risks and benefits, generally with antivirals administered to recipient • Vaccination recommended for all candidates
	Influenza Respiratory syncytial virus Human metapneumovirus Picornavirus, rhinovirus Parainfluenza Adenovirus	• Testing usually only performed in symptomatic donors/recipients, so true incidence and impact unknown • Some reports of DDI with poor outcomes • Recipients can be protected with vaccination against influenza and RSV • Antiviral therapy for influenza may reduce severity and infectivity
Mollicutes	*Ureaplasma urealyticum* *Ureaplasma parvum* *Mycoplasma hominis*	• Hyperammonemia syndrome associated with poor outcomes • Screening with cultures and/or NAT can identify cases before illness and facilitate pre-emptive treatment
Molds	*Aspergillus* spp. Other molds *Histoplasma*, *Blastomyces*, *Coccidioides*	• Lung donors can be exposed to mold infections • Antifungal prophylaxis or pre-emptive testing and targeted therapy can reduce impact • Endemic mycoses represent unique challenges in some regions, with screening and prophylaxis recommended for *Coccidioides* in donors/recipients from endemic areas, but not others
Mycobacteria	*Mycobacterium tuberculosis* Nontuberculous mycobacteria	• Donor-derived tuberculosis occasionally occurs; donor screening is not routine • Nontuberculous mycobacteria a less pathogenic and transmission is rare
**Heart transplant recipients**		
Endocarditis	Gram-positive organisms such as *Staphylococcus aureus* *G*ram negatives Occasionally yeasts	• Donor bacteremia common, heart utilization possible if no vegetations, normal myocardial function • Treatment of recipients with donor bacteremia with targeted antimicrobials recommended
Toxoplasmosis	*Toxoplasma gondii*	• Seronegative recipients receiving hearts from seropositive donors highest risk • Prophylaxis with trimethoprim-sulfamethoxazole highly effective
Chagas	*Trypanosoma cruzi*	• *T. cruzi* is endemic in Latin America and infection can result in significant cardiac complications • Screening of donors from endemic areas is recommended, avoiding heart donation if positive • Surveillance with PCR can be performed post-transplant, informing therapy with nifurtimox or benznidazole

Abbreviations: DDI, donor-derived infection; RSV, respiratory syncytial virus.

## Lung transplant

Lung transplant recipients require high levels of immunosuppression to prevent rejection and experience a high burden of infections. Comprehensive donor and recipient screening recommendations are in place and allow for identification of pathogens, risk stratification, and pre-emptive interventions.^3^ One of the unique aspects of lung transplantation compared to other solid organs is the fact that the lung is continually exposed to the external environment and is transplanted with an intact microbiome. This includes bacteria, viruses, and fungi from a variety of environmental exposures, including nosocomial acquisition before donation. Pre- and post-transplant bronchoscopy is standard of care for all potential donors and recipients, allowing microbiologic sampling to identify potential pathogens and inform prompt early treatment which can prevent progression to more invasive disease. Latent infections, such as tuberculosis (TB), remain elusive to diagnostics and can later reactivate in the setting of immunosuppression.

### Bacterial infections including multidrug resistance

Lung transplant recipients are at high risk for early bacterial infections, mainly of the respiratory tract, which can increase morbidity and mortality.^4^ Deceased donors are often in the intensive care unit for some time before donation, potentially exposing them to antibiotics and nosocomial pathogens including resistant organisms.^5^ Respiratory cultures are the most frequently positive donor cultures, but only relevant to lung transplant recipients.^6^ Perioperative antimicrobial prophylaxis targeting organisms present in both donor and recipient is standard of care and aims to prevent early pneumonia and improve outcomes.^7^, ^8^, ^9^ There is significant variation in prophylaxis regimens used worldwide and little data to inform practice, although many centers use 7 days for donors/recipients without colonization and 14 days if bacteria are present.^10^ Broad-spectrum antipseudomonal beta-lactam agents such as piperacillin-tazobactam or cefepime are frequently used empirically, with the choice and duration modified based on donor and recipient cultures. It is crucial to balance the benefits of antibiotics in reducing the burden of early bacterial infections against the risks of overuse of broad-spectrum agents driving further resistance and other side effects, especially in the absence of infection.

In the context of rising rates of antimicrobial resistance, particularly in gram-negative bacteria, there are increasing complexities in the choice of agents and decisions need to balance broader coverage of potentially resistant bacteria with the need to provide stewardship to avoid driving resistance. Many of the recognized transmissions of bacteria from donors to recipients are due to multidrug-resistant organisms (MDROs) resistant to perioperative antibiotics.

Donor MDROs are more likely to fail conventional prophylaxis and be transmitted to recipients, highlighting the importance of screening. In the Italian experience, carbapenem-resistant gram-negative pathogens were isolated from 18 donors, including 12 respiratory specimens, who proceeded to donate organs to 30 recipients, including 3 lung recipients.^11^ Transmission did not occur with targeted antimicrobials but occurred in 4 of 6 (67%) cases without this intervention, due to communication failures or risk underestimation. In another study, which included 119 lung recipients, carbapenem-resistant organisms (CRO) colonization was present in 4 lung donors, resulting in 3 transmissions.^12^ To address this, Italy has now implemented a local active surveillance system that manages MDRO transmission events, improving communication and facilitating optimized prophylaxis. A follow-up prospective cohort study of 600 deceased donors and 791 recipients (including 65 lung) identified 38 recipients with a high-risk CRO.^13^ All received timely, targeted prophylaxis, preventing transmission in 71% of patients, although only 3 of these were lung recipients. When DDI occurred, negative impacts were mitigated. These findings are supported by the Saudi experience, where donor MDROs have not been associated with worse outcomes in lung transplant recipients, and newer molecular techniques are being explored to expedite identification and optimize perioperative antimicrobial prophylaxis.^14^, ^15^, ^16^ Overall, these studies demonstrate that lung transplantation from donors colonized with MDROs, including CRO, is possible with communication of results and effective, targeted antimicrobial prophylaxis/pre-emptive therapy.^11^, ^13^, ^17^

### Respiratory viral infections

While lung transplant recipients remain uniquely vulnerable to more severe disease from respiratory viral infections, many are minimally symptomatic and have no significant sequelae. These viruses include influenza, respiratory syncytial virus (RSV), human metapneumovirus, picornavirus, rhinovirus, parainfluenza, adenovirus, and coronaviruses, including COVID-19. Any of these viruses could be present in a lung donor but given limited donor testing, the true incidence of respiratory viral infections is poorly defined. Early in the COVID-19 pandemic, testing of donors was not initially recommended and in this setting an untested, infected donor transmitted the infection to the recipient and to care-team members.^18^, ^19^, ^20^ As a result, most transplant programs have developed policies requiring lower airway sampling of donors for SARS-CoV-2 nucleic acid testing (NAT). With this testing strategy, transmissions have generally been avoided. However, as small amounts of SARS-CoV-2 RNA can be detected by NAT which may not represent viable infectious virions, this approach may result in loss of organs that could be safely transplanted.^21^, ^22^, ^23^ Routine testing is not generally recommended for other viruses, although it should be considered in symptomatic patients before becoming organ donors. Nonetheless, transmission of influenza, RSV, and human metapneumovirus has been demonstrated from infected donors to lung recipients.^24^, ^25^, ^26^, ^27^ It is well recognized that respiratory viral infections cause not only acute illness but can also contribute to chronic lung allograft dysfunction (CLAD) post-transplant.^28^ Studies are needed to understand if donor infection is associated with any increased risk of CLAD or other worse outcomes.

One approach to mitigate the risk of these DDIs is to ensure that candidates remain up-to-date with vaccines, particularly for influenza, RSV, and SARS-CoV-2. If organs are used and the donor is subsequently found to have SARS-CoV-2 or influenza, antiviral therapy can be considered and may mitigate against severe disease.^29^ Use of donors with documented respiratory viral infections, with perhaps the exception of rhinovirus or seasonal coronaviruses, is generally avoided for lung transplant unless the risk-benefit assessment is strongly in favor of use despite risk of disease; the candidate should be informed about the infection and the risk associated with it to participate in the decision. These organs can be safely utilized for non lung recipients as risk of transmission is generally low. Novel influenza viruses, such as influenza A/H5N1, do represent a risk of viremia and transmission and use of any organ from an infected donor should be avoided early in an outbreak.

### Mollicute infection

Hyperammonemia, a progressive elevation of ammonia most commonly found in lung transplant recipients, was historically associated with exceptionally poor outcomes. After initial recognition of an association between likely donor–derived *Ureaplasma* infection and subsequent development of hyperammonemia syndrome, further studies and reports have cemented the link between donor–derived mollicute infection causing the hyperammonemia syndrome, particularly in lung transplant recipients.^30^, ^31^, ^32^, ^33^, ^34^, ^35^, ^36^, ^37^, ^38^, ^39^, ^40^, ^41^, ^42^, ^43^, ^44^, ^45^, ^46^, ^47^ In one large study, 27 of 1,156 lung transplant recipients (2.3%) developed mollicute infection, which was associated with increased mortality.^43^

Mollicutes include *Mycoplasma* and *Ureaplasma* species. These bacteria lack a cell wall, grow poorly in routine culture, and utilize the cleavage of urea into ammonia as a component of their energy synthesis.^40^ *Ureaplasma* species are more dependent on this pathway for ATP synthesis. While there have been a few selected cases of hyperammonemia associated with *Mycoplasma*, most of the cases in the literature are associated with *U. urealyticum* or *U. parvum*. Screening can be performed with culture and/or NAT. NAT provides results much more quickly than cultures, but there are no commercial mollicute PCR assays approved for respiratory samples and testing may require sending to a specialized lab. While one study suggested culture to be more specific than NAT, most studies have found NAT testing to detect most cases, with the highest yield when both strategies are utilized.^42^ It is important to note that the rate of detection of mollicutes in donors is much higher than the rate of hyperammonemia detected clinically. *Mycoplasma* infection may be associated with surgical site or chest wall cavity infections and pre-emptive treatment following detection in a donor may not prevent all such infections.^48^

While routine donor testing is currently only required in Italy, the importance of early detection has now been well documented.^49^ When testing is performed late, particularly in the setting of ongoing elevations of ammonia, outcomes are worse than with routine early screening coupled with treatment. Some have advocated for serial ammonia testing in recipients but most cases of transient increases are not associated with mollicute infection and this approach may result in worse outcomes.^46^ Routine donor screening coupled with early empiric therapy reduces the risk of hyperammonemia and improves outcomes. If hyperammonemia is recognized, treatment should be initiated as soon as possible while awaiting confirmation of mollicute infection. Nearly all isolates are susceptible to tetracyclines, macrolides, and fluoroquinolones. Resistance to monotherapy has been described and current expert opinion recommends using at least 2 drugs for 14 days. The optimal combination and duration of therapy warrant further study. If screening results are available within 24 to 48 hours of transplant, treatment can be initiated if *Ureaplasma* is identified; if results take longer than 48 hours, some centers empirically treat and stop therapy if samples test negative.

### Mold infections

Invasive mold infections are common in lung transplant recipients and can also be donor-derived. Donor colonization with fungi is not uncommon, particularly in cases associated with trauma or soil/water exposure, such as drownings or motorbike accidents.^50^ Incidence in one cohort was around 4% but likely varies with donor epidemiology.^51^ Many centers use antifungal prophylaxis with either mold-active azoles such as voriconazole, posaconazole, or itraconazole, or inhaled amphotericin. Inhaled antifungals aim to minimize systemic toxicity while optimizing delivery to the affected site.^52^ Others use a pre-emptive screening approach based on bronchoscopic testing, allowing for identification of colonized individuals and early treatment initiation before progression to more invasive disease. Both strategies have advantages and disadvantages, and there is variation between centers based on local epidemiology and clinician preferences.

Endemic mycoses pose unique threats. Donors with confirmed histoplasmosis, blastomycosis, or coccidiomycosis should not be utilized but those with a history of treated or resolved endemic mycoses can be used with prophylaxis limited to those with coccidiomycosis. Reactivation of *Histoplasma* or *Blastomyces* is rare post-transplant and screening, even in endemic regions, is not routinely recommended.^53^ Coccidiomycosis, though, can relapse post-transplant, including among donors with prior exposures to endemic regions. Routine screening for coccidiomycosis of donors from and recipients in endemic regions is recommended with routine antifungal prophylaxis in those who test positive by serology or antigen.^54^, ^55^

### Mycobacterial infections

While rare, donor-derived TB can occur and may be associated with poor outcomes, particularly in lung transplant recipients.^56^, ^57^, ^58^, ^59^, ^60^, ^61^ Current guidelines recommend against the use of donors with known active TB for any organ.^1^, ^58^ Transmission can occur with all organ types and atypical presentations may occur; the lung recipient may not be the index patient identified with donor-transmitted TB. If transmission is suspected or confirmed, early initiation of standard therapy for active TB consistent with local guidelines is recommended.^59^

While screening for latent TB in transplant candidates, typically now with interferon-gamma release assays, is standard, testing of donors has been poorly studied and is not routinely performed.^58^ Very few studies have explored Quantiferon-Gold testing in donors; one study of 38 deceased donors in Iran found that 3 (8%) were positive (consistent with local prevalence), 24 (63%) were negative, and 11 (29%) were indeterminate, likely due to donor energy.^62^ All donors should be assessed for risk factors, which in most cases of donor–derived TB transmission can be identified. A retrospective study of potential donor–derived TB transmission events in the United States from 2008-2018 involving 51 unique deceased donors and 156 recipients described proven/probable transmissions in 9 donors/35 recipients, and possible transmission in 18 donors/71 recipients.^63^ Many donors had risk factors for TB, lungs were the most affected organ recipient group (6 proven/probable, 10 possible), and prophylaxis was administered successfully to many patients. Most centers perform routine surveillance bronchoscopy at regular intervals in the first year post-transplant with mycobacterial cultures sent, enabling early diagnosis if reactivation of latent donor infection occurs.^59^ If a case of suspected donor-derived TB is identified in any organ recipient, rapid communication via the organ procurement organization is crucial to enable timely assessment and treatment of other recipients.^64^ Although rare, if a donor is known to have untreated latent TB, the recipient should receive latent TB treatment as soon post-transplant as possible.

While nontuberculous mycobacteria are a challenge to manage post-transplant, there have not been reports of donor–derived nontuberculous mycobacteria transmission. This may be in part due to lack of testing of lung donors routinely. These organisms are typically less pathogenic and treatment of colonization is not usually required, with the exception of *Mycobacterium abscessus*.

## Heart transplant

Heart transplant recipients are overall lower risk for DDI, but some specific risks arise because it is the only transplanted organ that is a muscle and is also exposed to the bloodstream in a unique way, increasing risk of endovascular infection. Here, we will focus on 3 scenarios: donor–derived endocarditis, toxoplasmosis, and Chagas disease.

### Donor bacteremia and endocarditis

Donor bloodstream infections are relatively common and represent a transmission risk for any organ recipient.^65^, ^66^, ^67^, ^68^ An array of community and nosocomial pathogens can be seen, including gram positives such as *Staphylococcus aureus*, gram negatives, and occasionally yeasts.^26^ When potential donors are found to have bacteremia, follow-up blood cultures should be collected and antibiotics commenced without delay. Donation can generally proceed once pathogen identification and susceptibilities are known, with antibiotics administered for at least 24 to 48 hours before organ procurement where possible. Final results can be used to determine ongoing recipient treatment. Choice and duration of antimicrobial therapy is typically targeted antibiotics for 14 days post-transplant, although pathogen-dependent and optimally guided by transplant infectious diseases physicians considering all relevant factors.

Donor-derived endocarditis is a rare but potentially serious complication of heart transplantation. The International Society for Heart and Lung Transplantation guidelines recommend that hearts from donors with serious infections, including known bacteremia, can be utilized provided certain criteria are met, including the administration of pathogen-directed antimicrobials to both donor and recipient, normal myocardial function, and the absence of evidence of vegetations or other evidence of endocarditis on direct inspection of donor heart valves before implantation.^69^ Because recipients have been treated with antibiotics routinely in this way for many years to mitigate risk of bacterial transmission, donor-derived endocarditis is a rare event. However, cases have been reported, more often with MDROs, after shorter or suboptimal courses of antibiotics were given.^68^, ^70^

### Toxoplasmosis

*Toxoplasma gondii* is a ubiquitous parasite that can be found worldwide, with an estimated seroprevalence of 30% to 50% which varies widely geographically.^71^ Infection is acquired from ingestion of raw or undercooked meat, inhalation of cat feces, or contaminated soil. Infection persists long-term, and immune control is required to prevent reactivation. Acquisition from blood transfusion and solid organ transplantation is also well reported. Because the cysts of *T. gondii* persist in muscle tissue, seronegative recipients receiving hearts from seropositive donors (D+/R−) are at the highest risk. Before the introduction of routine screening and prophylaxis, transmission was commonly seen in this subgroup often resulting in clinical disease with substantial morbidity and mortality.^72^, ^73^ Screening of all donors and recipients is now recommended with IgG testing; donor testing is required by the Organ Procurement and Transplantation Network (OPTN) in the United States.^58^

Trimethoprim-sulfamethoxazole prophylaxis is highly effective at preventing toxoplasma reactivation and is now in widespread use.^74^, ^75^, ^76^ The optimal duration is unclear and many heart recipients receive prophylaxis for greater than a year. Allergies or tolerances to trimethoprim-sulfamethoxazole may result in the use of suboptimal prophylaxis and should be carefully evaluated to ensure they represent a true contraindication, with desensitization a potential option for some patients. Second-line *Pneumocystis* preventative agents such as nebulized pentamidine and dapsone have no activity against *T. gondii* so reactivation can occur while taking these agents unless pyrimethamine is added. Atovaquone has some activity, but breakthrough cases have occurred, predominantly in bone marrow transplant recipients.^77^, ^78^, ^79^

### Chagas disease

*Trypanosoma cruzi* is endemic to large parts of Latin America, with a seroprevalence of up to 8% in some regions, highest in Central America.^80^ Given the global migration of individuals from endemic regions, donor-derived Chagas from donors who previously lived in higher risk areas has been seen in places such a the United States and Spain where testing is not necessarily routine. Infection is transmitted by the reduviid bugs of the *Triatominae* subfamily, which are blood-sucking insects also known as “kissing bugs” that excrete infectious trypomastigotes in their feces.^81^ When they defecate after feeding, infection can occur through the bite wound or mucosal surfaces.

While acute infection can be associated with fever, malaise, lymphadenopathy, hepatosplenomegaly, or an erythematous swelling at the site of entry known as a chagoma, many patients are asymptomatic.^81^ In the absence of treatment with nifurtimox or benznidazole, which can be difficult to access and have associated toxicities, chronic infection develops which can also be asymptomatic or have cardiac and/or gastrointestinal manifestations. An array of cardiac complications can develop, including arrhythmias and heart failure.

While there is a potential for DDI with any transplanted organ, heart transplant is the highest risk due to chronic infection of the myocardium. DDIs are well reported, including a series of 32 recipients from 14 *T. cruzi* seropositive donors who transmitted to 9 recipients, including 3 of 4 (75%) heart recipients.^82^ Screening of donors from endemic areas is recommended with serology, avoiding heart transplantation from positive donors.^83^, ^84^ Seropositive donors or recipients should trigger routine post-transplant screening, ideally with PCR, for early detection of reactivation. Initiation of therapy can prevent symptoms of infection and the heightened risk of rejection with parasitemia.^85^, ^86^ Without screening, patients may present with myocarditis manifested with arrhythmia and conduction abnormalities, valve insufficiency, and graft dysfunction with heart failure or with extracardiac manifestations, including fever skin lesions, and, rarely, meningoencephalitis.^87^

## Other donor-derived infections

### Bloodborne viruses

The bloodborne virus landscape in solid organ transplantation has changed substantially around the world over the last decade or so. Most jurisdictions now perform routine nucleic acid testing for HIV, hepatitis B and C in addition to serology on all donors, which significantly decreases the likelihood of DDI by reducing the window/eclipse period to 11 to 13 days for HIV, 20 to 22 days for hepatitis B, and 3 to 5 days for hepatitis C.^88^ This has made the identification of donor risk factors less relevant. The introduction of highly effective direct-acting antiviral therapy for hepatitis C has enabled post-transplant treatment and utilization of donors with active infection, increasing the pool with excellent outcomes.^89^ The 2013 HIV Organ Policy Equity Act permitted transplantation from HIV positive donors to HIV positive recipients in the United States and has resulted in many successful transplants.^90^ Transplantation from nonviremic donors with a positive hepatitis core antibody is safe and standard of care, with antiviral prophylaxis and vaccination mitigating the risk of transmission. Hepatitis B surface antigen-positive donors have been explored as a potential option to increase the donor pool.^91^, ^92^, ^93^, ^94^, ^95^

In 2020, there were updates made to the US Public Health Service guidance with recognition of these advances.^88^ There were several recommendations made by an expert committee, which included moving away from the terminology labeling donors “high-risk” or “increased-risk.” This nomenclature could result in an excessive perception of risk from recipient centers and patients, potentially resulting in declined offers leading to worse outcomes.^96^, ^97^ Outcomes from these donors have been good, with disease transmissions rare in the presence of modern preventative strategies.^98^

## Conclusions

Donor-derived infections remain an important challenge in heart and lung transplant recipients, where some unique risks are present. Although rare overall, these infections are associated with worse outcomes, and many are predictable and preventable. Measures such as screening with serology or other tests, risk stratification, and implementation of antimicrobial prophylaxis can reduce the incidence of these infections (Table 2). Recognition of clinical syndromes when they occur, prompt diagnosis and effective treatment, with rapid and effective communication via organ procurement organizations to enable earlier intervention in other recipients, is of key importance. Together, these measures can improve patient outcomes and allow transplant recipients to live longer, healthier lives.

**Table 2:** Donor Screening Tests Recommended in Heart and Lung Transplantation

Test	Population	Recommended actions if positive
Donor medical and social history	All donors	Identify risk factors to guide further testing
Blood culture	All donors	Antimicrobials to recipient for 7-14 days, guided by bacterial identification, susceptibilities, site and clinical setting
Respiratory cultures for bacteria, mycobacteria, and fungi (BAL)	Lung donors only	Targeted antimicrobials to recipient for 7-14 days
BAL *Ureaplasma* PCR and/or culture	Lung donors only	Targeted antimicrobials to recipient for 14 days
BAL SARS-CoV-2 PCR	Lung donors only	Carefully consider risks vs benefits of proceeding. Antivirals (remdesivir) for recipient. Other non lung organs can be used safely with negligible risk
Human immunodeficiency virus serology and NAT	All donors	Can be used with specific informed consent, usually restricted to HIV positive recipients in select centers
Hepatitis B serology and NAT	All donors	Antiviral prophylaxis and/or monitoring of recipient
Hepatitis C serology and NAT	All donors	Antiviral treatment for recipient
Cytomegalovirus serology	All donors	Risk stratification, antiviral prophylaxis, virologic monitoring
Epstein-Barr virus serology	All donors	Risk stratification, clinical and virologic monitoring
Syphilis screening or diagnostic assay	All donors	Treatment of recipient
Toxoplasma serology	All donors	Risk stratification (particularly heart recipients), prophylaxis
Coccidiomycosis serology	Donors from endemic regions	Extended courses of antifungal prophylaxis
Chagas serology	Donors from endemic regions	Pre-emptive monitoring of recipient, treatment if positive
West Nile virus PCR	Donors during seasonal circulation	Transplantation contraindicated

Abbreviations: BAL, bronchoalveolar lavage.

## Author contributions

B.G. and M.I. both participated in preparing this manuscript. B.G. focused on the literature review and manuscript writing, while M.I. provided supervision and oversight. Both authors have reviewed the final manuscript and agree with its content.

## Disclosure statement

The authors declare that they have no known competing financial interests or personal relationships that could have appeared to influence the work reported in this paper.

B.G. has received support from the 10.13039/501100001232Royal Australasian College of Physicians Research Establishment Fellowship.

## References

[1] Wolfe C.R., Ison M.G. (2019). Practice ASTIDCo: donor-derived infections: guidelines from the American Society of Transplantation Infectious Diseases Community of Practice. Clin Transplant.

[2] Prakash K., Saharia K.K., Karaba A. (2024). Minimizing risk while maximizing opportunity: the infectious disease organ offer process survey. Transpl Infect Dis.

[3] Trachuk P., Bartash R., Abbasi M., Keene A. (2020). Infectious complications in lung transplant recipients. Lung.

[4] Shields R.K., Clancy C.J., Minces L.R. (2013). Epidemiology and outcomes of deep surgical site infections following lung transplantation. Am J Transplant.

[5] Fischer S.A. (2019). Is this organ donor safe?: Donor-derived infections in solid organ transplantation. Surg Clin North Am.

[6] Boutin C.A., Pouch S.M., Ison M.G. (2023). Utility of deceased donor cultures in solid organ transplantation in preventing donor-derived bacterial and fungal infectious diseases transmission. Transpl Infect Dis.

[7] Abbo L.M., Grossi P.A. (2019). Practice AICo: surgical site infections: guidelines from the American Society of Transplantation Infectious Diseases Community of Practice. Clin Transplant.

[8] Anesi J.A., Blumberg E.A., Abbo L.M. (2018). Perioperative antibiotic prophylaxis to prevent surgical site infections in solid organ transplantation. Transplantation.

[9] Bratzler D.W., Dellinger E.P., Olsen K.M. (2013). Clinical practice guidelines for antimicrobial prophylaxis in surgery. Am J Health Syst Pharm.

[10] Coiffard B., Prud'Homme E., Hraiech S. (2020). Worldwide clinical practices in perioperative antibiotic therapy for lung transplantation. BMC Pulm Med.

[11] Mularoni A., Bertani A., Vizzini G. (2015). Outcome of transplantation using organs from donors infected or colonized with carbapenem-resistant gram-negative bacteria. Am J Transplant.

[12] Errico G., Gagliotti C., Monaco M. (2019). Colonization and infection due to carbapenemase-producing *Enterobacteriaceae* in liver and lung transplant recipients and donor-derived transmission: a prospective cohort study conducted in Italy. Clin Microbiol Infect.

[13] Mularoni A., Cona A., Campanella M. (2024). Donor-derived carbapenem-resistant gram-negative bacterial infections in solid organ transplant recipients: active surveillance enhances recipient safety. Am J Transplant.

[14] Abdulqawi R., Saleh R.A., Alameer R.M. (2024). Donor respiratory multidrug-resistant bacteria and lung transplantation outcomes. J Infect.

[15] Abdulqawi R., Saleh R.A., Alameer R.M. (2025). Universal multiplex panel testing of donor lungs as a strategy to optimize antibiotic prophylaxis against multidrug-resistant bacteria. Transpl Infect Dis.

[16] Lombardi A., Mangioni D., Renisi G., Liparoti A., Bandera A. (2025). Re: Universal multiplex panel testing of donor lungs as a strategy to optimize antibiotic prophylaxis against multidrug-resistant bacteria. Transpl Infect Dis.

[17] Pouch S.M., Patel G. (2019). Practice ASTIDCo: multidrug-resistant gram-negative bacterial infections in solid organ transplant recipients-guidelines from the American Society of Transplantation Infectious Diseases Community of Practice. Clin Transplant.

[18] Kaul D.R., Valesano A.L., Petrie J.G. (2021). Donor to recipient transmission of SARS-CoV-2 by lung transplantation despite negative donor upper respiratory tract testing. Am J Transplant.

[19] Shah M.B., Lynch R.J., El-Haddad H., Doby B., Brockmeier D., Goldberg D.S. (2020). Utilization of deceased donors during a pandemic: argument against using SARS-CoV-2-positive donors. Am J Transplant.

[20] Kates O.S., Fisher C.E., Rakita R.M., Reyes J.D., Limaye A.P. (2020). Use of SARS-CoV-2-infected deceased organ donors: should we always “just say no?”. Am J Transplant.

[21] Eichenberger E.M., Coniglio A.C., Milano C. (2022). Transplanting thoracic COVID-19 positive donors: an institutional protocol and report of the first 14 cases. J Heart Lung Transplant.

[22] Kothadia S.M., Wolfe C.R., Baker A.W. (2025). Outcomes of lung transplantation from SARS-CoV-2 positive donors during the Omicron wave. JHLT Open.

[23] Asija R., Singh R., Paneitz D.C. (2023). Is transplantation with coronavirus disease 2019-positive donor lungs safe? A US nationwide analysis. Ann Thorac Surg.

[24] Meylan P.R., Aubert J.D., Kaiser L. (2007). Influenza transmission to recipient through lung transplantation. Transpl Infect Dis.

[25] Le Page A.K., Kainer G., Glanville A.R., Tu E., Bhonagiri D., Rawlinson W.D. (2010). Influenza B virus transmission in recipients of kidney and lung transplants from an infected donor. Transplantation.

[26] Kaul D.R., Vece G., Blumberg E. (2021). Ten years of donor-derived disease: a report of the disease transmission advisory committee. Am J Transplant.

[27] Hulot S., Prunet V., Coiffard B. (2025). Fatal acute respiratory distress syndrome caused by donor-derived human metapneumovirus after lung transplantation. Am J Transplant.

[28] Gardiner B.J., Snydman D.R. (2016). Editorial commentary: chronic lung allograft dysfunction in lung transplant recipients: another piece of the puzzle. Clin Infect Dis.

[29] Ennis S.L., Levvey B.J., Shingles H.V., Lee S.J., Snell G.I., Gardiner B.J. (2024). COVID-19 infection is mild and has minimal impact on lung function in well vaccinated and widely treated lung transplant recipients. J Heart Lung Transplant.

[30] Bharat A., Cunningham S.A., Scott Budinger G.R. (2015). Disseminated *Ureaplasma* infection as a cause of fatal hyperammonemia in humans. Sci Transl Med.

[31] Bharat A., Scott Budinger G.R., Ison M.G. (2017). Donor-derived *Ureaplasma* is a potentially lethal infection in lung allograft recipients. J Heart Lung Transplant.

[32] Buzo B.F., Preiksaitis J.K., Halloran K. (2021). Association between *Mycoplasma* and *Ureaplasma* airway positivity, ammonia levels, and outcomes post-lung transplantation: a prospective surveillance study. Am J Transplant.

[33] Cheema F., Kutzler H.L., Olowofela A.S. (2020). Successful management of noncirrhotic hyperammonemia syndrome after kidney transplantation from putative *Ureaplasma* infection. Transpl Infect Dis.

[34] Divithotewala C., Sweeney E.L., Burke A. (2023). *Mycoplasma hominis* and *Ureaplasma urealyticum* infections in the immediate post-lung transplant period: a case series and literature review. Transpl Infect Dis.

[35] Farfour E., Vasse M., Vallee A. (2024). Mollicutes-related infections in thoracic surgery including lung and heart transplantation: a systematic review. J Heart Lung Transplant.

[36] Fernandez R., Ratliff A., Crabb D., Waites K.B., Bharat A. (2017). Ureaplasma transmitted from donor lungs is pathogenic after lung transplantation. Ann Thorac Surg.

[37] Kurihara C., Manerikar A., Kandula V., Cerier E., Bharat A. (2021). Prophylactic *Ureaplasma*-directed antimicrobials in lung donors can prevent fatal hyperammonemia. Transplantation.

[38] Lum J.M., Roberts S.C. (2022). Hyperammonemia in lung transplantation: recognizing the gaps of our knowledge. Transpl Infect Dis.

[39] Roberts S.C., Bharat A., Kurihara C., Tomic R., Ison M.G. (2021). Impact of screening and treatment of ureaplasma species on hyperammonemia syndrome in lung transplant recipients: a single center experience. Clin Infect Dis.

[40] Roberts S.C., Malik W., Ison M.G. (2022). Hyperammonemia syndrome in immunosuppressed individuals. Curr Opin Infect Dis.

[41] Schwartz S., Aldrich A., Kessler E., Abulaban K., Steinke J.M. (2023). Disseminated *Ureaplasma* polyarthritis in a renal transplant recipient. Pediatr Transplant.

[42] Tam P.C.K., Alexander B.D., Lee M.J. (2024). Donor-derived *Mycoplasma* and *Ureaplasma* infections in lung transplant recipients: a prospective study of donor and recipient respiratory tract screening and recipient outcomes. Am J Transplant.

[43] Tam P.C.K., Hardie R., Alexander B.D. (2024). Risk factors, management, and clinical outcomes of invasive *Mycoplasma* and *Ureaplasma* infections after lung transplantation. Am J Transplant.

[44] Vecchio M., Koutsokera A., Touilloux B. (2021). Bronchial anastomosis dehiscence and stenosis caused by donor-transmitted *Mycoplasma hominis* infection in a lung transplant recipient: case report and literature review. Transpl Infect Dis.

[45] Vijayvargiya P., Esquer Garrigos Z., Kennedy C.C. (2022). Routine donor and recipient screening for *Mycoplasma hominis* and *Ureaplasma* species in lung transplant recipients. Open Forum Infect Dis.

[46] Walti L.N., Ng C.F., Kaur S. (2025). Serum ammonia screening and donor mollicute detection for hyperammonemia syndrome post-lung transplant: a prospective observational study. Clin Infect Dis.

[47] Wigston C., Lavender M., Long R. (2023). *Mycoplasma* and *Ureaplasma* donor-derived infection and hyperammonemia syndrome in 4 solid organ transplant recipients from a single donor. Open Forum Infect Dis.

[48] Smibert O.C., Wilson H.L., Sohail A. (2017). Donor-derived *Mycoplasma hominis* and an apparent cluster of *M. hominis* cases in solid organ transplant recipients. Clin Infect Dis.

[49] Grossi P.A., Lombardini L., Donadio R., Peritore D., Feltrin G. (2024). Perspective on donor-derived infections in Italy. Transpl Infect Dis.

[50] Wu K., Annambhotla P., Free R.J. (2023). Fatal invasive mold infections after transplantation of organs recovered from drowned donors, United States, 2011-2021. Emerg Infect Dis.

[51] Mularoni A., Cona A., Coniglione G. (2024). Donor-derived mold infections in lung transplant recipients: the importance of active surveillance. Transpl Infect Dis.

[52] Gardiner B.J., Ivulich S.P., Snell G.I. (2024). Voriconazole inhalation powder: a novel therapeutic alternative for invasive pulmonary fungal infections. Transpl Infect Dis.

[53] Singh N., Huprikar S., Burdette S.D. (2012). Donor-derived fungal infections in organ transplant recipients: guidelines of the American Society of Transplantation, infectious diseases community of practice. Am J Transplant.

[54] Lee D.H., Abidi M.Z., Fisher C. (2025). Coccidioidomycosis transmission through solid organ transplantation (2013-2022): a report of the Organ Procurement and Transplantation Network ad hoc Disease Transmission Advisory Committee. Transpl Infect Dis.

[55] Lohrmann G.M., Vucicevic D., Lawrence R. (2017). Single-center experience of antifungal prophylaxis for coccidioidomycosis in heart transplant recipients within an endemic area. Transpl Infect Dis.

[56] Kay A., Barry P.M., Annambhotla P. (2017). Solid organ transplant-transmitted tuberculosis linked to a community outbreak - California, 2015. MMWR Morb Mortal Wkly Rep.

[57] Singh N., Paterson D.L. (1998). *Mycobacterium tuberculosis* infection in solid-organ transplant recipients: impact and implications for management. Clin Infect Dis.

[58] Malinis M., Boucher H.W. (2019). Practice ASTIDCo: Screening of donor and candidate prior to solid organ transplantation-guidelines from the American Society of Transplantation Infectious Diseases Community of Practice. Clin Transplant.

[59] Morris M.I., Daly J.S., Blumberg E. (2012). Diagnosis and management of tuberculosis in transplant donors: a donor-derived infections consensus conference report. Am J Transplant.

[60] Mortensen E., Hellinger W., Keller C. (2014). Three cases of donor-derived pulmonary tuberculosis in lung transplant recipients and review of 12 previously reported cases: opportunities for early diagnosis and prevention. Transpl Infect Dis.

[61] Kumar D., Budev M., Koval C., Hellinger W.C., Gordon S.M., Tomford J.W. (2013). Donor-derived tuberculosis (TB) infection in lung transplant despite following recommended algorithm. Am J Transplant.

[62] Tabarsi P., Yousefzadeh A., Najafizadeh K. (2014). Performance of QuantiFERON TB Gold test in detecting latent tuberculosis infection in brain-dead organ donors in Iran: a brief report. Saudi J Kidney Dis Transpl.

[63] Malinis M., La Hoz R.M., Vece G. (2022). Donor-derived tuberculosis among solid organ transplant recipients in the United States-2008 to 2018. Transpl Infect Dis.

[64] Clemente W.T., Faria L.C., Cota G.F. (2021). Donor-derived tuberculosis: a case report and the role of communication gaps in transplantation safety. Case Rep Transplant.

[65] Freeman R.B., Giatras I., Falagas M.E. (1999). Outcome of transplantation of organs procured from bacteremic donors. Transplantation.

[66] Kovacs C.S., Koval C.E., van Duin D. (2014). Selecting suitable solid organ transplant donors: reducing the risk of donor-transmitted infections. World J Transplant.

[67] Lumbreras C., Sanz F., Gonzalez A. (2001). Clinical significance of donor-unrecognized bacteremia in the outcome of solid-organ transplant recipients. Clin Infect Dis.

[68] Sifri C.D., Ison M.G. (2012). Highly resistant bacteria and donor-derived infections: treading in uncharted territory. Transpl Infect Dis.

[69] Velleca A., Shullo M.A., Dhital K. (2023). The International Society for Heart and Lung Transplantation (ISHLT) guidelines for the care of heart transplant recipients. J Heart Lung Transplant.

[70] Wendt J.M., Kaul D., Limbago B.M. (2014). Transmission of methicillin-resistant *Staphylococcus aureus* infection through solid organ transplantation: confirmation via whole genome sequencing. Am J Transplant.

[71] La Hoz R.M., Morris M.I., Infectious Diseases Community of Practice of the American Society of Transplantation (2019). Tissue and blood protozoa including toxoplasmosis, Chagas disease, leishmaniasis, Babesia, Acanthamoeba, Balamuthia, and Naegleria in solid organ transplant recipients - guidelines from the American Society of Transplantation Infectious Diseases Community of Practice. Clin Transplant.

[72] Gallino A., Maggiorini M., Kiowski W. (1996). Toxoplasmosis in heart transplant recipients. Eur J Clin Microbiol Infect Dis.

[73] Fernandez-Sabe N., Cervera C., Farinas M.C. (2012). Risk factors, clinical features, and outcomes of toxoplasmosis in solid-organ transplant recipients: a matched case-control study. Clin Infect Dis.

[74] Baran D.A., Alwarshetty M.M., Alvi S. (2006). Is toxoplasmosis prophylaxis necessary in cardiac transplantation? Long-term follow-up at two transplant centers. J Heart Lung Transplant.

[75] Baden L.R., Katz J.T., Franck L. (2003). Successful toxoplasmosis prophylaxis after orthotopic cardiac transplantation with trimethoprim-sulfamethoxazole. Transplantation.

[76] Munoz P., Arencibia J., Rodriguez C. (2003). Trimethoprim-sulfamethoxazole as toxoplasmosis prophylaxis for heart transplant recipients. Clin Infect Dis.

[77] Garcia de la Fuente I., Ansari M., Rougemont A.L. (2010). Acute disseminated fatal toxoplasmosis after haploidentical stem cell transplantation despite atovaquone prophylaxis in a young man. Pediatr Infect Dis J.

[78] Mendorf A., Klyuchnikov E., Langebrake C. (2015). Atovaquone for prophylaxis of toxoplasmosis after allogeneic hematopoietic stem cell transplantation. Acta Haematol.

[79] Petty L.A., Qamar S., Ananthanarayanan V. (2015). Cardiac toxoplasmosis after heart transplantation diagnosed by endomyocardial biopsy. Transpl Infect Dis.

[80] Moncayo A., Silveira A.C. (2009). Current epidemiological trends for Chagas disease in Latin America and future challenges in epidemiology, surveillance and health policy. Mem Inst Oswaldo Cruz.

[81] Swett M.C., Rayes D.L., Campos S.V., Kumar R.N. (2024). Chagas disease: epidemiology, diagnosis, and treatment. Curr Cardiol Rep.

[82] Huprikar S., Bosserman E., Patel G. (2013). Donor-derived *Trypanosoma cruzi* infection in solid organ recipients in the United States, 2001-2011. Am J Transplant.

[83] Chin-Hong P.V., Schwartz B.S., Bern C. (2011). Screening and treatment of Chagas disease in organ transplant recipients in the United States: recommendations from the Chagas in transplant working group. Am J Transplant.

[84] Kransdorf E.P., Zakowski P.C., Kobashigawa J.A. (2014). Chagas disease in solid organ and heart transplantation. Curr Opin Infect Dis.

[85] Barcan L., Radisic M. (2025). Don't wait for the storm-fix the roof while the sun shines! Lessons for ensuring safe transplantation in patients at risk for Chagas Disease. Transpl Infect Dis.

[86] Oliveira J.F., Barbosa A.L., Cota G. (2025). Reactivation of Chagas disease in the central nervous system among heart transplant recipients: a clinical series from Brazil, 2006-2023. Transpl Infect Dis.

[87] Radisic M.V., Repetto S.A. (2020). American trypanosomiasis (Chagas disease) in solid organ transplantation. Transpl Infect Dis.

[88] Jones J.M., Kracalik I., Levi M.E. (2020). Assessing solid organ donors and monitoring transplant recipients for human immunodeficiency virus, hepatitis B virus, and hepatitis C virus infection - U.S. Public Health Service Guideline, 2020. MMWR Recomm Rep.

[89] Steinbrink J.M., Byrns J., Berg C. (2023). Real-world experiences in the transplantation of hepatitis C-NAAT-positive organs. Transplant Direct.

[90] Werbel W.A., Brown D.M., Kusemiju O.T. (2022). National landscape of human immunodeficiency virus-positive deceased organ donors in the United States. Clin Infect Dis.

[91] Huprikar S., Danziger-Isakov L., Ahn J. (2015). Solid organ transplantation from hepatitis B virus-positive donors: consensus guidelines for recipient management. Am J Transplant.

[92] Carnemolla B.T., Kutzler H.L., Kuzaro H.A. (2022). Use of hepatitis B viremic donors in kidney transplant recipients: a single center experience. Transpl Infect Dis.

[93] Yost C.C., Jimenez D.C., Weber M.P. (2023). Hepatitis B in heart transplant donors and recipients: a systematic review and meta-analysis. J Surg Res.

[94] Chancharoenthana W., Townamchai N., Pongpirul K. (2014). The outcomes of kidney transplantation in hepatitis B surface antigen (HBsAg)-negative recipients receiving graft from HBsAg-positive donors: a retrospective, propensity score-matched study. Am J Transplant.

[95] Chancharoenthana W., Leelahavanichkul A. (2021). Kidney transplantation from hepatitis B surface antigen (HBsAg)-positive living donors to HBsAg-negative recipients: benefits and risks. Clin Infect Dis.

[96] Volk M.L., Wilk A.R., Wolfe C., Kaul D.R. (2017). The “PHS Increased Risk” label is associated with nonutilization of hundreds of organs per year. Transplantation.

[97] Limkemann A.J., Wolfe L., Sharma A. (2016). Outcomes of kidney transplants and risk of infection transmission from increased infectious risk donors. Clin Transplant.

[98] Grossi P.A., Wolfe C., Peghin M. (2024). Non-standard risk donors and risk of donor-derived infections: from evaluation to therapeutic management. Transpl Int.

